# PopHuman: the human population genomics browser

**DOI:** 10.1093/nar/gkx943

**Published:** 2017-10-20

**Authors:** Sònia Casillas, Roger Mulet, Pablo Villegas-Mirón, Sergi Hervas, Esteve Sanz, Daniel Velasco, Jaume Bertranpetit, Hafid Laayouni, Antonio Barbadilla

**Affiliations:** Institut de Biotecnologia i de Biomedicina and Department de Genètica i de Microbiologia, Universitat Autònoma de Barcelona, 08193 Bellaterra, Barcelona, Spain; Institute of Evolutionary Biology (UPF-CSIC), Universitat Pompeu Fabra, Doctor Aiguader 88 (PRBB), 08003 Barcelona, Catalonia, Spain; Servei de Genòmica i Bioinformàtica, Universitat Autònoma de Barcelona, 08193 Bellaterra, Barcelona, Spain; Bioinformatics Studies, ESCI-UPF, Pg. Pujades 1, 08003 Barcelona, Spain

## Abstract

The 1000 Genomes Project (1000GP) represents the most comprehensive world-wide nucleotide variation data set so far in humans, providing the sequencing and analysis of 2504 genomes from 26 populations and reporting >84 million variants. The availability of this sequence data provides the human lineage with an invaluable resource for population genomics studies, allowing the testing of molecular population genetics hypotheses and eventually the understanding of the evolutionary dynamics of genetic variation in human populations. Here we present PopHuman, a new population genomics-oriented genome browser based on JBrowse that allows the interactive visualization and retrieval of an extensive inventory of population genetics metrics. Efficient and reliable parameter estimates have been computed using a novel pipeline that faces the unique features and limitations of the 1000GP data, and include a battery of nucleotide variation measures, divergence and linkage disequilibrium parameters, as well as different tests of neutrality, estimated in non-overlapping windows along the chromosomes and in annotated genes for all 26 populations of the 1000GP. PopHuman is open and freely available at http://pophuman.uab.cat.

## INTRODUCTION

Soon after the elucidation of the entire human genome (1–3), the description of genetic variation in human populations and the identification of those variants that affect health and disease became the next challenges of genomics research (4). The International HapMap Consortium built the first genome-wide catalog of common human genetic variation in diverse populations (4–6), charting haplotype maps of 1.6 million single nucleotide polymorphisms (SNPs) in 1184 reference individuals from 11 global populations. In addition to numerous genome-wide association studies (GWAS) (7), the HapMap data allowed the detection of positive natural selection across the human genome (8,9), as well as the development of new tests to infer recent episodes of selective sweeps based on the length of haplotypes, such as the Long-Range Haplotype (LRH) (10), the integrated Haplotype Score (iHS) (11), and the Cross Population Extended Haplotype Homozygosity (XP-EHH) (8).

During the last decade, the development of next generation sequencing (NGS) technologies (12,13) has allowed the deciphering of complete genome sequences of thousands of human individuals, and the 1000 Genomes Project (1000GP) has become the reference data set for population genetics and genomics (14,15). With the aim of providing a deep characterization of human genome sequence variation, the most recent version of the 1000GP (Phase III) completes the sequencing and analysis of 2504 genomes from 26 populations and describes most variants with frequencies as low as 1%. Due to its higher resolution and smaller SNP ascertainment bias compared to HapMap genotyping data, the availability of the 1000GP data provides the human lineage with an invaluable resource on which to test molecular population genetics hypotheses and eventually understand the evolutionary dynamics of genetic variation in human populations (16).

Regions of the genome that are (or have been) subject to natural selection show distinctive patterns of genetic variation in the DNA sequence (17). The signature of long-range haplotypes persists for a relatively short period of time (<30 000 years), and related statistics can detect very recent selection only. However, other signatures persist longer in the genome: differentiation between populations (<50 000–<75 000 years), high frequency derived alleles (<80 000 years), reduction in genetic diversity and excess of rare alleles (<250 000 years), and high proportion of function-altering substitutions between species (many millions of years) (17).

Population genomics analyses of the 1000GP data set can be largely facilitated by (i) making an inventory of parameter values along the chromosomes that capture the evolutionary properties of the available sequences, and (ii) allowing the query and visualization of these estimates in a genome browser designed specifically for this data. As far as we are concerned, the 1000 Genomes Selection Browser 1.0 (18) is the only previous database that allows the interactive visualization and retrieval of population genetics metrics for the 1000GP data. It was published when the 1000GP was still in its first phase (1,092 individuals, 14 populations, 38 million SNPs) (14), and analyzed within-species polymorphism data for three populations in 30 kb sliding windows (18). Here, we present PopHuman, a new population genomics-oriented genome browser. PopHuman represents not only an update to the 1000GP Phase III (2504 individuals, 26 populations, 84.7 million SNPs), but also dramatic improvements in the amount of data analyzed and browser performance, compared to the 1000 Genomes Selection Browser 1.0. Furthermore, PopHuman analyzes between-species divergence, which allows the implementation of statistical tests to detect the signature of recurrent natural selection acting over prolonged periods of time, such as the McDonald and Kreitman test (MKT) (19), instead of recent selective sweeps only. Supplementary Table S1 details the differences between the two databases.

## POPHUMAN ANALYSIS PIPELINE

**Figure 1.**
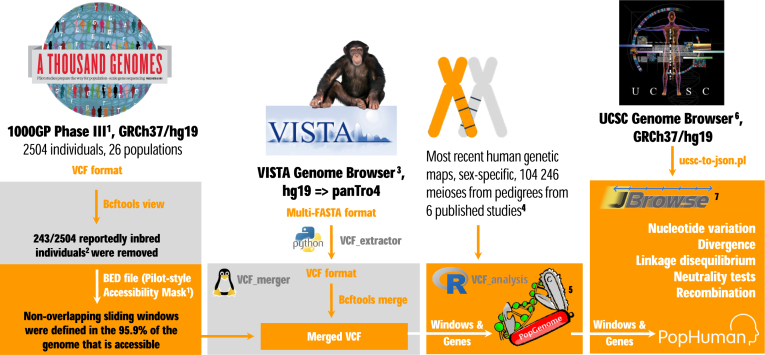
PopHuman pipeline. Cited references in the figure: **^1^**1000GP Phase III (15); **^2^**Inbred individuals in the 1000GP (20); **^3^**VISTA Genome Browser (23); **^4^**Human genetic maps (24); **^5^**PopGenome software (25); **^6^**UCSC Genome Browser (35); **^7^**JBrowse software (34).

We have designed and implemented a custom pipeline (Figure 1) facing the unique features and limitations of the 1000GP Phase III data (15). The pipeline discards reportedly inbred individuals (20) and non-accessible nucleotides (15), incorporates the genomic sequence of the chimpanzee (21) as outgroup, and estimates a battery of nucleotide variation, divergence and linkage disequilibrium parameters, as well as different tests of neutrality, on the filtered data. Several metrics have been computed both in non-overlapping sliding windows along the chromosomes and in annotated protein coding genes for 26 populations of distinct geographical origin (15).

### Pre-processing of the 1000GP Phase III data

We retrieved human genome variation data generated by the 1000GP Phase III (15) from http://www.internationalgenome.org/data in Variant Call Format (VCF). This included 84.4 million variants detected across 2504 individuals from 26 different populations, mapped to the human reference genome version GRCh37/hg19. We want to warn the user that four of the analyzed populations present admixture (corresponding to the Admixed American metapopulation), so special care should be taken while interpreting PopHuman results in those cases.

#### Inbred individuals

The initial VCF files were filtered to exclude 243 individuals with inbreeding coefficients similar or greater than the ones expected for first-cousin offspring, according to Gazal *et al.* (20).

#### Genome accessibility mask

Due to the nature of short-read sequencing, sequencing depth varies along the length of the genome. The 1000GP provides an ‘accessibility mask’, a Browser Extensible Data (BED) file that indicates which sites of the genome were accessible to the sequencing techniques and have power for variant discovery (15). Two definitions were used in the Phase III, of which we selected the ‘pilot-style’ mask. This definition is less conservative than the ‘strict’ mask while being still adequate for population genomics analyses, and was chosen to maximize the amount of genomic sequence to be analyzed. It excludes the portion of the genome where depth of coverage (summed across all samples) was higher or lower than the average depth by a factor of 2-fold, as well as sites where >20% of overlapping reads had mapping quality of zero. Overall, 89.4% of the genome is considered reliable (95.9% of the non-N bases). Specifically, we placed 10 kb non-overlapping sliding windows in accessible regions of the genome (i.e. windows do not overlap any non-accessible nucleotide) to focus on high quality genomic regions only. Table 1 summarizes the total amount of data analyzed by PopHuman by following this methodology. In addition, we analyzed longer non-overlapping sliding windows of 100 kb placed all along the genome (i.e. windows might overlap non-accessible nucleotides, although these positions were discarded for the population genomics analyses) to focus on broader scale patterns of diversity across the genome.

**Table 1.** Summary of the amount of data analyzed in PopHuman

Chromosome		Windows-based analysis		Genes-based analysis	
Chromosome number	Chromosome size (millions of bases)^a^	Number of windows^b^	Number of bases (millions)	Percentage of analyzed bases	Number of RefSeq^c^ genes analyzed
**1**	249.25	14 741	147.41	59.14	2328
**2**	243.20	16 270	162.70	66.90	1464
**3**	198.02	13 575	135.75	68.55	1274
**4**	191.15	12 512	125.12	65.45	879
**5**	180.92	12073	120.73	66.73	1022
**6**	171.12	11 433	114.33	66.81	1206
**7**	159.14	9919	99.19	62.33	1108
**8**	146.36	9783	97.83	66.84	818
**9**	141.21	7358	73.58	52.11	944
**10**	135.53	8760	87.60	64.63	903
**11**	135.01	8877	88.77	65.75	1439
**12**	133.85	8773	87.73	65.54	1175
**13**	115.17	6481	64.81	56.27	449
**14**	107.35	5948	59.48	55.41	779
**15**	102.53	5334	53.34	52.02	791
**16**	90.35	4688	46.88	51.88	938
**17**	81.20	4556	45.56	56.11	1358
**18**	78.08	5164	51.64	66.14	341
**19**	59.13	2681	26.81	45.34	1609
**20**	63.03	4091	40.91	64.91	647
**21**	48.13	2211	22.11	45.94	296
**22**	51.30	2009	20.09	39.16	535
**X**	155.27	9312	93.12	59.97	918
**Y**	59.37	622	6.22	10.48	53
**TOTAL**	3095.68	187 171	1871.71	60.46	23 274

^a^Chromosome sizes are according to version GRCh37/hg19 of the human genome.

^b^Non-overlapping sliding windows of 10 kb have been defined such that they do not include non-accessible bases according to the Pilot-style Accessibility Mask of the 1000GP (15).

^c^RefSeq genes provided by the NCBI Entrez Gene database (33).

#### Ancestral states

The ancestral states of human segregating sites were taken from the 1000GP Phase III (15), which were obtained by using the 6-way EPO alignments available in Ensembl v71 (22).

#### Outgroup species

To compute divergence metrics and neutrality tests based on the comparison of polymorphism and divergence, we added differences between humans and chimpanzees to the VCF files, as identified from a precomputed hg19 => panTro4 alignment obtained from the VISTA browser (23) in multi-FASTA format (MFA). Specifically, the pairwise alignment was converted to VCF using custom scripts and merged with the 1000GP VCF files using *bcftools merge*.

### Recombination

The most recent human genetic sex-specific maps were obtained from Bhérer *et al.* (24), based on a total of 104 246 informative meioses from six recent studies of human pedigrees.

### Estimation of population genomics statistics

#### Windows-based

Several windows-based variation statistics and tests of neutrality (Table 2) were computed for each population separately using the R package PopGenome (25) and custom functions, considering biallelic SNPs as within-species variation data. Haplotype-based statistics (iHS and XP-EHH) were computed in a multithreaded framework implemented by the program selscan (26), considering biallelic SNPs with Minor Allele Frequency (MAF) > 0.05 and a maximum gap of 20 kb between two consecutive SNPs. Then, whole chromosome per-SNP scores were summarized by calculating the mean of the absolute value of these scores for all SNPs in a window (27). Sexual chromosomes were not analyzed in these cases.

**Table 2.** List of major windows-based variation statistics and tests of neutrality in PopHuman, computed for each population separately

Category	Track name	Track description	Reference
**Frequency-based nucleotide variation**	S	Number of segregating sites per site	(42)
	Pi	Nucleotide diversity: average number of nucleotide differences per site between any two sequences	(42–44)
	theta	Nucleotide polymorphism: proportion of nucleotide sites that are expected to be polymorphic in any suitable sample	(45–47)
	hap_diversity_within	Haplotype diversity within the population	(48)
**Divergence-based metrics**	Divsites	Number of divergent sites	
	K	Nucleotide divergence per base pair, corrected by Jukes-Cantor	(43)
**Linkage disequilibrium**	Kelly_ZnS	Average pairwise *r*^2^ value	(49)
	Rozas_ZA	Average of *r*^2^ only between adjacent polymorphic sites	(50)
	Rozas_ZZ	Rozas_ZA minus Kelly_ZnS	(50)
	Wall_B; Wall_Q	Proportion of pairs of adjacent segregating sites that are congruent, with values approaching 1 indicating extensive congruence among adjacent segregating sites	(51)
	iHS	Integrated haplotype score, based on the frequency of alleles in regions of high LD (computed for the autosomes)	(11)
	XP_EHH	Long-range haplotype method to detect recent selective sweeps (computed for the autosomes, between the major continental populations CEU, CHB and YRI, taken in pairs)	(8)
**Recombination**	recomb_Bherer2017_females/males/sexavg	Recombination estimates (cM/Mb) from the refined genetic map by Bhérer *et al.* (2017), which collects recombination events from six recent studies of human pedigrees, pertaining to a total of 104 246 informative meioses. Maps are available in three separate tracks: females, males and sexavg	(24)
	recomb_deCODE_ females/males/sexavg	deCODE genetic map based on 5136 microsatellite markers for 146 families with a total of 1257 meiotic events.	(52)
	recomb_Marshfield_ females/males/sexavg	Marshfield genetic map based on 8325 short tandem repeat polymorphisms (STRPs) for 8 CEPH families consisting of 134 individuals with 186 meioses.	(53)
	recomb_Genethon_ females/males/sexavg	Genethon genetic map based on 5264 microsatellites for 8 CEPH families consisting of 134 individuals with 186 meioses.	(54)
**Selection tests based on SFS and/or variability**	FayWu_H	Number of derived nucleotide variants at low and high frequencies with the number of variants at intermediate frequencies	(55)
	FuLi_D	Number of derived nucleotide variants observed only once in a sample with the total number of derived nucleotide variants	(29)
	FuLi_F	Number of derived nucleotide variants observed only once in a sample with the mean pairwise difference between sequences	(29)
	Tajima_D	Difference between the number of segregating sites and the average number of nucleotide differences.	(56)
	Zeng_E	Difference between θ_L_ and θ_W_, sensitive to changes in high-frequency variants.	(57)
**Selection tests based on the MKT**	DoS	Direction of Selection: difference between the proportion of nonsynonymous divergence and nonsynonymous polymorphism	(58)
	NI	Neutrality Index: summarizes the four values in a McDonald and Kreitman test table as a ratio of ratios	(19,59)
	alpha; alpha_cor	Proportion of substitutions that are adaptive. The second is calculated after removing slightly deleterious mutations	(19,32,60,61)

*A complete list is available under the section* Help → Tracks Description *of PopHuman*.

#### Genes-based

Comparisons of DNA polymorphism within populations and divergence to an outgroup species using the MKT (19) have been extensively used to detect the signature of natural selection at the molecular level (28). The MKT can be generalized to any two types of sites provided that one of them is assumed to evolve neutrally and that both types of sites are closely linked in the genome (29–31). Furthermore, Mackay *et al.* (32) developed an integrative new framework for the MKT by incorporating information on the MAF of the segregating sites, which allows estimating the fraction of new mutations that are strongly deleterious (and therefore not segregating), slightly deleterious (segregating at low frequency), old neutral (neutral before the split of humans and chimpanzees), and recently neutral (since the split of humans and chimpanzees), as well as the fraction of adaptive fixations. The standard and integrative MKTs (Table 3) were applied to all annotated human protein coding genes in RefSeq (33) and for different types of sites (i.e. 0-fold nonsynonymous coding sites, 5′UTR, 3′UTR, introns, and ±500 bp intergenic flanking regions, compared to 4-fold synonymous coding sites), for each population separately, using custom functions build within PopGenome (25).

**Table 3.** List of major gene-based variation statistics in PopHuman, computed for each population separately and for different types of sites

Category	Estimate	Description	Reference	Types of sites analyzed
**Descriptive statistics**	π	Nucleotide diversity: average number of nucleotide differences per site between any two sequences	(42–44)	Whole gene region ±500 bp
	K	Nucleotide divergence per base pair, corrected by Jukes-Cantor	(43)	
	π_a_/π_s_	Ratio of nonsynonymous to synonymous nucleotide polymorphism (ω)	(44,62)	Ratio: 0-fold divided by 4-fold
	K_a_/K_s_	Ratio of nonsynonymous to synonymous nucleotide divergence (ω)	(44,62)	
	DAF	Derived Allele Frequency: distribution of allele frequencies of segregating sites	(63)	Whole gene region ±500 bp
**Recombination (Bhérer *et al.* 2017), cM/Mb**	cM/Mb	Recombination estimates (cM/Mb) from the refined genetic map by Bhérer *et al.* 2017	(24)	Whole gene region ±500 bp
**Standard MKT**	P	Number of segregating sites	(42)	Separately: 4-fold; 0-fold; 5′UTR; 3′UTR; intron; intergenic (±500 bp)
	D	Number of divergent sites		
	π	Nucleotide diversity: average number of nucleotide differences per site between any two sequences	(42–44)	
	K	Nucleotide divergence per base pair, corrected by Jukes-Cantor	(43)	
	α	Proportion of substitutions that are adaptive. It is calculated both from P and D, and from π and K	(19,32,60,61)	
**Integrative MKT**	*d*	Fraction of new mutations that are strongly deleterious and do not segregate in the population	(32)	Separately: 0-fold; 5′UTR; 3′UTR; intron; intergenic (±500 bp)
	*b*	Fraction of new mutations that are slightly deleterious and segregate at minor allele frequency (MAF) <5%		
	ƒ-γ	Fraction of new mutations that are neutral since before the split of humans and chimpanzees, calculated after removing the excess of sites at MAF <5% due to slightly deleterious mutations		
	γ	Fraction of new mutations that have become neutral recently, after the split of humans and chimpanzees, calculated after removing the excess of sites at MAF <5% due to slightly deleterious mutations		
	α	Proportion of substitutions that are adaptive, calculated after removing slightly deleterious mutations	(19,32,60,61)	
	DoS	Direction of Selection: difference between the proportion of nonsynonymous divergence and nonsynonymous polymorphism	(58)	

A comprehensive explanation is available under the section Help → Integrative MKT of PopHuman.

## OVERVIEW OF THE POPHUMAN GENOME BROWSER

PopHuman is a new population genomics-oriented genome browser based on JBrowse (34) that allows the interactive visualization and retrieval of several metrics estimated in non-overlapping sliding windows along the chromosomes and in annotated genes for all 26 populations of the 1000GP. It also includes a number of utilities and support resources.

### JBrowse implementation

PopHuman is built on JBrowse (34) and is currently running under Apache on a CentOS 7.2 Linux x64 server with 16 Intel Xeon 2.4 GHz processors and 32 GB RAM.

### Browser tracks

#### Variation statistics

Windows-based variation statistics and tests of neutrality (Table 2) are classified into: (i) frequency-based nucleotide variation; (ii) divergence-based metrics; (iii) linkage disequilibrium; (iv) recombination; (v) selection tests based on the Site Frequency Spectrum (SFS) and/or variability and (vi) selection tests based on the MKT. They are displayed for each population separately as histogram plots, with a yellow line showing the mean, and two shaded bands showing ±1 and ±2 standard deviations from the mean. Visualization style can be customized using the ‘Edit config’ option for each track.

#### Reference tracks

Several tracks have been imported from the UCSC Genome Browser (35) (Supplementary Table S2) and can be visualized along with variation statistics. They are classified into: (i) sequencing and annotation; (ii) regulation; (iii) comparative genomics; (iv) variation and (v) repeats.

### Utilities and support resources

#### Tracks selector

PopHuman contains more than a thousand tracks, including both variation statistics (Table 2) and reference tracks (Supplementary Table S2). Given the large number of tracks available, these can be filtered and selected using the ‘Select tracks’ tool, which can be accessed from the top left corner, below the navigation bar. The filtering process is normally performed by first narrowing the search using the menu on the left, and then selecting the tracks of interest from the main panel on the right. This process can be done several times in order to finally get all the desired tracks selected.

#### Downloading raw data

Variation statistics for a given region can be conveniently downloaded in bedGraph, Wiggle or GFF3 formats using the ‘Save track data’ option for each track. In addition, bulk downloads of full variation tracks are available in BigWig format from the Resources menu. Finally, variant calls for the analyzed individuals can also be downloaded in VCF format using the PopHuman utility ‘Download sequences’, which can be accessed either from the Resources menu, or directly from the navigation bar.

#### Integrative MKT

Gene-based MKTs (Table 3) can be retrieved by right-clicking a gene and selecting the option ‘Integrative MKT’.

#### Help section

The Help section contains exhaustive documentation about the 1000GP Phase III data analyzed by PopHuman and details about the browser tracks. Interestingly, it contains a comprehensive tutorial introducing to the usage of the database and to the testing of evolutionary hypotheses from a population genetics perspective. The tutorial works out, in different sequential steps, the visualization and analysis of a genomic region of around 20 kb in chromosome 7 that includes the *TRPV6* gene. *TRPV6* is a well-studied protein coding gene involved in the absorption of calcium from the diet that has experienced parallel selective sweeps in non-African populations, coinciding with the establishment of agriculture first in Europe around 10 000 years ago, and later in Asia. The tutorial contains several step-by-step guides to facilitate reproducing the results that are shown both in the form of figures and descriptive text.

### Availability

All data, tools and support resources provided by PopHuman, as well as reference tracks downloaded from the UCSC Genome Browser (35), are open and freely available at http://pophuman.uab.cat.

## COMPARISON TO OTHER DATABASES

While the PopHuman analysis pipeline presented here is completely novel, the genome browser is based on a similar instance previously developed by our group that hosts population genomics statistics for 30 *Drosophila melanogaster* populations (36). Novel features that have been implemented in PopHuman include the utility to retrieve gene-based integrative MKT metrics.

Compared to the 1000 Genomes Selection Browser 1.0 (18), PopHuman presents three significant advantages. First, PopHuman analyzes the 1000GP Phase III data, which included 2.29 times more sampled sequences (2504 versus 1092) compared to the Phase I, and used an improved variant calling pipeline. Specifically, Phase III implemented an expanded set of variant callers, including some that use haplotype information and others that rely on *de novo* assembly, it considered low coverage and exome sequencing data jointly rather than independently, and used a different genotype calling that allowed the integration of multi-allelic variants and complex events (15). Second, PopHuman analyzes 26 instead of just three populations. This allows detecting very recent selective sweeps that have occurred in a single population and that can only be detected by analyzing data for this specific population; or older selective sweeps shared among a few related populations, whose detection gives a reinforcement of the time depth and biology underlying the specific selection signal. Three illustrative examples are provided: (i) a recent selective sweep related to skin pigmentation (37) in the region comprising the genes *SLC24A5, MYEF2, SLC12A1* and *CTXN2* in European (EUR) and South Asian (SAS) populations but not in East Asian (EAS) populations (Supplementary Figure S1); (ii) the presence of high frequency derived alleles in the gene *TRPV6* in all non-African populations, with a stronger signature in EAS populations, intermediate in SAS populations, and weaker in EUR populations, reflecting the time frame in which the establishment of agriculture, and thus the corresponding selective sweeps, occurred in those populations (stronger signatures in more recent sweeps; Supplementary Figure S2) and (iii) the presence of high frequency derived alleles in the Duffy red cell antigen gene (*DARC, FY, ACKR1*) in sub-Saharan Africa, thought to be the result of selection for resistance to *P. vivax* malaria (38,39), which is also seen in EAS populations (Supplementary Figure S3). Finally, PopHuman, contrary to the 1000 Genomes Selection Browser 1.0, implements selection tests based on the comparison of polymorphism and divergence, which are the only ones able to reveal the fixation of adaptive variants and other signatures of recurrent selection occurring over the last millions of years. One extreme example is found in the gene *PRM1*, which encodes a sperm-specific protein that compacts sperm DNA and shows a clear excess of function-altering substitutions between humans and chimpanzees compared to synonymous substitutions, indicative of positive Darwinian selection (40,41) (Supplementary Figure S4).

## CONCLUSION

The PopHuman database and browser go a step forward in the description and analysis of the most comprehensive human diversity data to date from a population genomics perspective. We aim PopHuman to be extended to incorporate novel metrics of transcriptomic and epigenomic variation, not only across individuals and species but also during the lifetime of an individual and/or in different parts of the body. In this way, PopHuman will become a pioneer population multi-omics browser advancing the upcoming population –omics synthesis (16).

## Supplementary Material

Supplementary Data
