# Supplementary material for: PopHuman: the human population genomics browser
Source: Nucleic Acids Res. 2017 Oct 20;46(Database issue):D1003–10. doi: 10.1093/nar/gkx943 (PMC5753332; doi:10.1093/nar/gkx943)
Supplement: Supplementary Data [file gkx943_supp.pdf]

PopHuman: the human population genomics browser

Sònia Casillas☯, Roger Mulet☯, Pablo Villegas-Mirón, Sergi Hervas, Esteve Sanz, Daniel Velasco, Jaume Bertranpetit, Hafid Laayouni, and Antonio Barbadilla

SUPPLEMENTARY DATA

Table S1. From the 1000G Selection Browser 1.0 to PopHuman. Links are provided to different sections of the PopHuman Help.

The 1000 Genomes Selection Browser 1.0	PopHuman
Source data is the **1000GP Phase I**: 1,092 individuals, 14 populations, 38 million SNPs.	Source data is the **1000GP Phase III**: 2,504 individuals, 26 populations, 84.7 million SNPs [Data description].
Analyses are performed for 3 populations: CEU, CHB, YRI.	Analyses are performed for 26 populations: 5 European populations, 7 African populations, 5 East-Asian populations, 5 South-Asian populations, and 4 Ad-mixed American populations [Data description].
Contains and analyzes within-species polymorphism data. Calculated statistics include nucleotide polymorphism, linkage disequilibrium, neutrality tests and population differentiation.	In addition to polymorphism data, PopHuman contains and analyzes between-species divergence data (with chimpanzee). Calculated statistics include nucleotide polymorphism and divergence, linkage disequilibrium, and neutrality tests based on the Site Frequency Spectrum as well as on polymorphism and divergence (standard and integrative MKT) [Tracks description]. Among others, this allows us to estimate which is the fraction of mutations that are strongly deleterious, weakly deleterious, neutral before the split of humans and chimpanzees, or recently neutral (since the split of humans and chimpanzees) [Integrative MKT].
Analyses are performed on 30 kb sliding windows.	Analyses are performed on 10 kb and 100 kb sliding windows, as well as for each annotated RefSeq gene separately, where different functional regions (0-fold nonsynonymous coding sites, 5'UTR, 3'UTR, introns, and ±500 bp intergenic flanking regions) are tested for the action of natural selection against 4-fold synonymous coding sites. Gene-by-gene analyses are available by right clicking a gene and selecting the option "Integrative MKT" [Integrative MKT].
It is composed by a set of tracks uploaded to an instance of the UCSC Genome Browser.	More than 1000 tracks, a convenient tracks filtering tool, gene-by-gene analyses, plus other useful utilities are all encapsulated in an agile web browser based on Jbrowse.
The site points to the genomic regions of some examples of selective sweeps in the human genome, providing a specific summary of literature and statistical tests in each case.	The site contains a comprehensive tutorial introducing to the usage of PopHuman and to the testing of evolutionary hypotheses from a population genetics perspective [PopHuman Tutorial]. The tutorial works out, in different sequential steps, the visualization and analysis of a genomic region of around 20 kb in chromosome 7 that includes the *TRPV6* gene. *TRPV6* is a well-studied protein coding gene involved in the absorption of calcium from the diet that has experienced parallel selective sweeps in non-African populations, coinciding with the establishment of agriculture first in Europe around 10,000 years ago, and later in Asia.

Table S2. Reference tracks imported from the UCSC Genome Browser. Links are provided for each track to its corresponding description in the UCSC.

Category	Track name	Track description
**Sequencing and annotation**	**Gene annotations**	Gene annotations in the human hg19 reference genome. This track is a set of gene predictions based on data from RefSeq, GenBank, CCDS, Rfam, and the tRNA Genes track. It includes both protein-coding genes and non-coding RNA genes. Annotations in this track are linked to the NCBI and UCSC databases. [Track information]
	**Reference Sequence**	Reference sequence of the human hg19 genome. [Track information]
	**Alignability of 36mers by GEM from ENCODE/CRG(Guigo)**	Measures how often the sequence found at a particular location (36mers) aligns within the whole genome. It tolerates up to 2 mismatches. Ranges from 0 to 1. [Track information]
	**Gaps**	Gaps in the assembly represented as black boxes. [Track information]
	**Mappability DAC Blacklisted Regions from ENCODE/DAC(Kundaje)**	Identifies regions of the reference genome that are troublesome for high throughput sequencing aligners. Troubled regions may be due to repetitive elements or other anomalies. [Track information]
	**Uniqueness of 35bp Windows from ENCODE/OpenChrom (Duke)**	Measures sequence uniqueness throughout the reference genome. Ranges from 0 to 1. [Track information]
**Regulation**	**Conserved Transcription Factor Binding Sites (TFBSs)**	This track contains the location and score of TFBSs conserved in the human/mouse/rat alignment. [Track information]
	**CpG Islands**	This track shows CpG islands that are associated with genes, particularly housekeeping genes, in vertebrates. CpG islands are regions where CpGs are present at significantly higher levels than is typical for the genome as a whole. CpG islands in repeats are masked. [Track information]
	**ORegAnno**	This track displays literature-curated regulatory regions, transcription factor binding sites, and regulatory polymorphisms from ORegAnno (Open Regulatory Annotation). [Track information]
	**Vista Enhancers**	The VISTA Enhancer Browser identifies distant-acting transcriptional enhancers in the human genome by coupling the identification of evolutionary conserved non-coding sequences with a moderate throughput mouse transgenesis enhancer assay. [Track information]
**Comparative genomics**	**100 vertebrates Basewise Conservation by PhyloP**	This track shows multiple alignments of 100 vertebrate species and measurements of evolutionary conservation using phyloP from the PHAST package, for all species. [Track information]
	**100 vertebrates Conserved Elements (phastConsElements100way)**	This track shows the conserved elements obtained using PhastCons. The predicted elements are segments of the alignment that are likely to have been "generated" by the conserved state of the phylo-HMM. [Track information]
	**100 vertebrates conservation by PhastCons**	This track shows multiple alignments of 100 vertebrate species and measurements of evolutionary conservation using PhastCons from the PHAST package, for all species. [Track information]

Category	Track name	Track description
**Comparative genomics** *(continued)*	**Genomic Evolutionary Rate Profiling (GERP)**	GERP is a method for producing position-specific estimates of evolutionary constraint using maximum likelihood evolutionary rate estimation. It also discovers "constrained elements" where multiple positions combine to give a signal that is indicative of a putative functional element; this track shows the position-specific scores only, not the element predictions. [Track information]
**Variation**	**1000 Genomes Project Phase 3 Paired-end Accessible Regions - Pilot Criteria**	This track shows which genome regions are more or less accessible to next generation sequencing methods that use short, paired-end reads. Pilot stringency regions cover 94.5% of non-N bases in the genome. [Track information]
	**1000 Genomes Project Phase 3 Paired-end Accessible Regions - Strict Criteria**	This track shows which genome regions are more or less accessible to next generation sequencing methods that use short, paired-end reads. Strict regions cover 75.5% (76.9% on autosomes). Each site meeting the Strict criteria also passes the Pilot criteria. [Track information]
	**DGV Struct Var Database of Genomic Variants: Structural Var Regions (CNV, Inversion, In/del)**	This track displays copy number variants (CNVs), insertions/deletions (InDels), inversions and inversion breakpoints annotated by the Database of Genomic Variants (DGV), which contains genomic variations observed in healthy individuals. [Track information]
	**Simple Nucleotide Polymorphisms (dbSNP 147)**	This track contains information about single nucleotide polymorphisms and small insertions and deletions (indels), from dbSNP build 147. [Track information]
**Repeats**	**Repeating Elements: RepeatMasker**	This track shows a detailed annotation of the repeats that are present in the query sequence. [Track information]
	**Segmental Dups**	This track shows regions detected as putative genomic duplications (>1 kb, >90% similar) within the golden path. [Track information]
	**Simple Tandem Repeats (STRs)**	This track displays simple tandem repeats (possibly imperfect repeats) located by Tandem Repeats Finder (TRF), which is specialized for this purpose. [Track information]

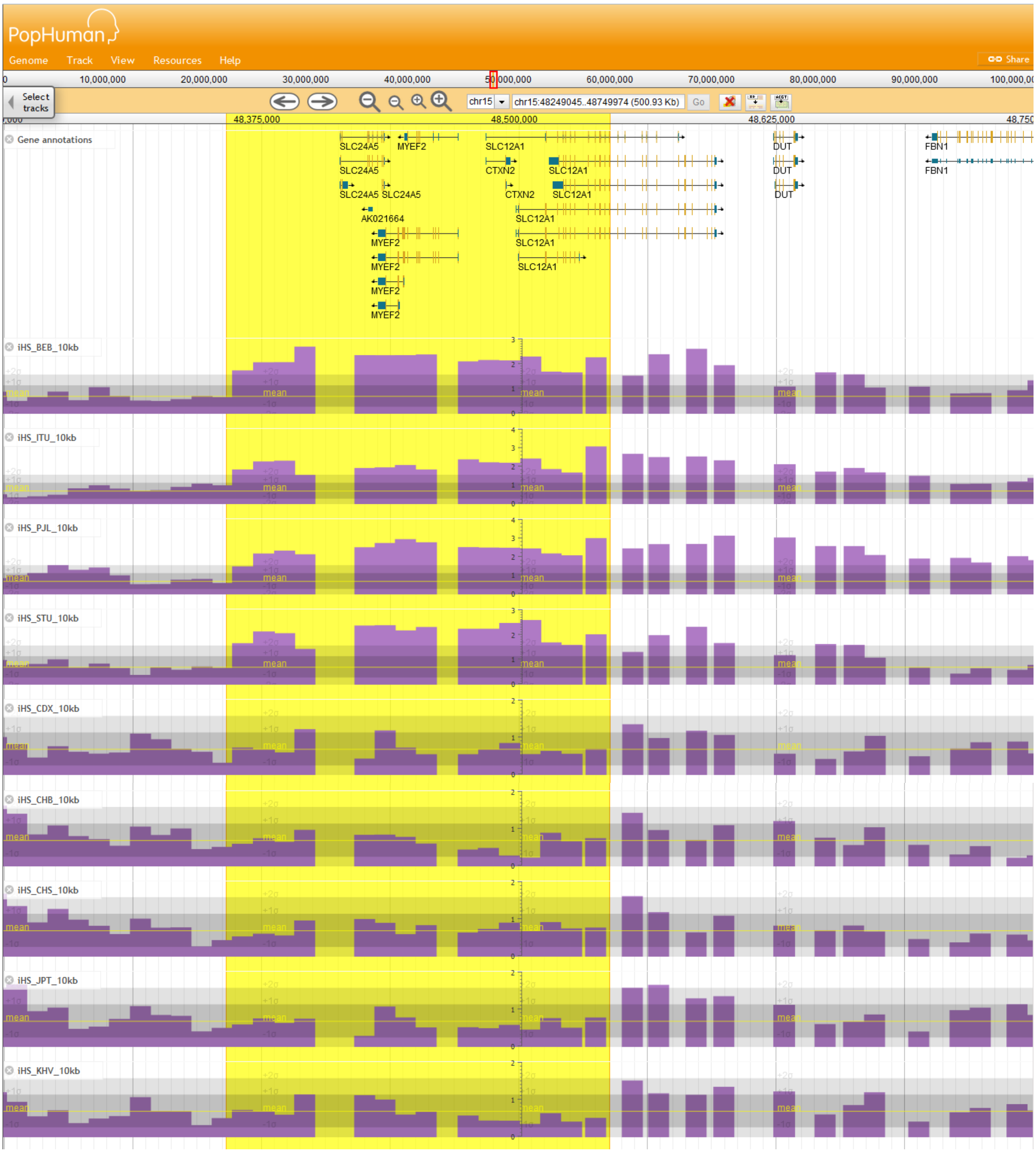

Figure S1. Screenshot of PopHuman showing the region comprising the genes *SLC24A5*, *MYEF2*, *SLC12A1*, and *CTXN2*, with iHS tracks activated for South Asian (SAS) and East Asian (EAS) populations. Extreme values of iHS in the first four tracks, corresponding to SAS populations, are indicative of a recent selective sweep related to skin pigmentation that occurred less than 30,000 years ago, which is the time that long haplotypes can persist in the genome after the sweep occurred. The signature is not shared with EAS populations (last five tracks).

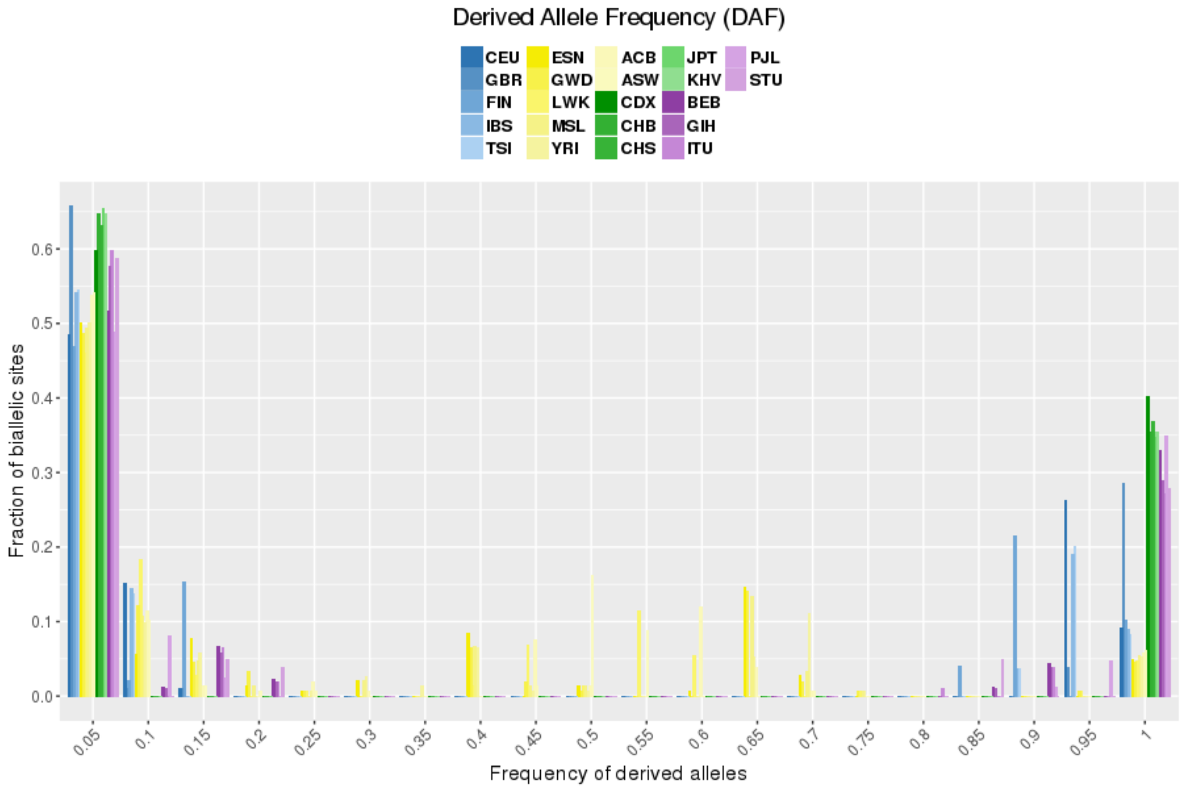

Figure S2. Screenshot of PopHuman showing the Derived Allele Frequency spectrum for the gene *TRPV6* in European (EUR, blue), African (AFR, yellow), East Asian (EAS, green), and South Asian (SAS, purple) populations. The gene is involved in the absorption of calcium from the diet and has experienced parallel selective sweeps in non-African populations, coinciding with the establishment of agriculture first in Europe around 10,000 years ago, and later in Asia. All non-AFR populations show an excess of high frequency derived alleles, with a stronger signature in EAS populations, intermediate in SAS populations, and weaker in EUR populations, reflecting the time frame in which the establishment of agriculture, and thus the corresponding selective sweeps, occurred in those populations (stronger signatures in more recent sweeps).

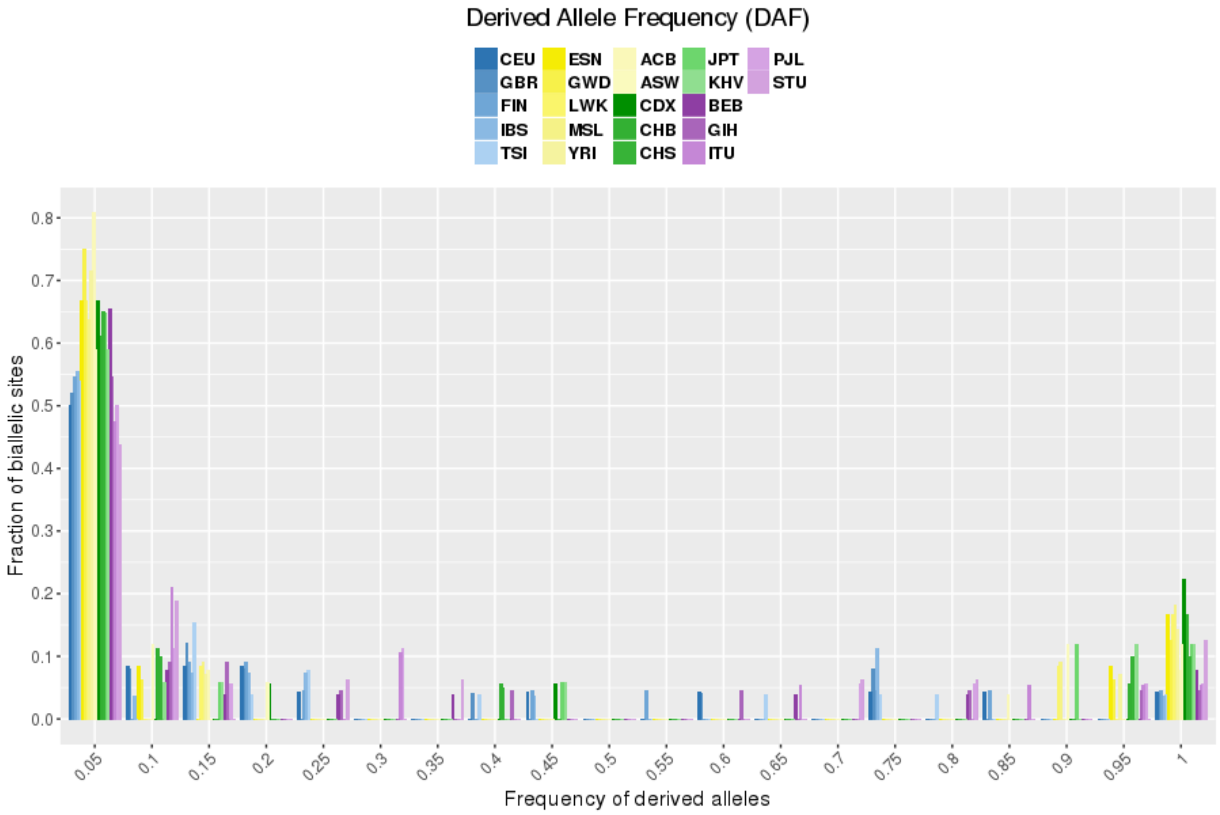

Figure S3. Screenshot of PopHuman showing the Derived Allele Frequency spectrum for the Duffy red cell antigen gene (*DARC*, *FY*, *ACKR1*) in European (EUR, blue), African (AFR, yellow), East Asian (EAS, green), and South Asian (SAS, purple) populations. AFR populations show an excess of high frequency derived alleles, thought to be the result of selection for resistance to *P. vivax* malaria. Surprisingly, a similar signature is found in EAS populations, while it is weaker in SAS populations and not present in EUR populations.

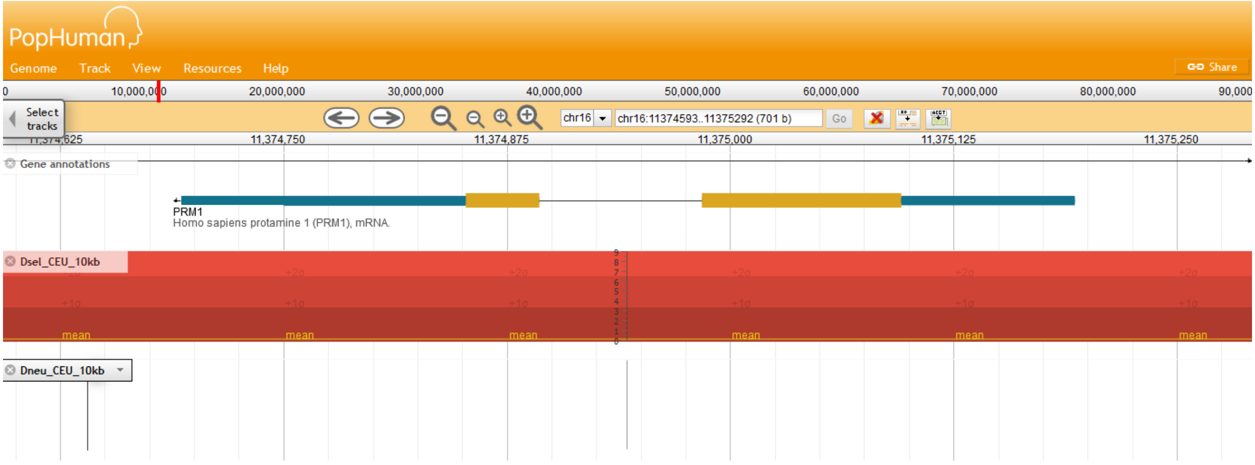

Figure S4. Screenshot of PopHuman showing the region of the gene *PRM1*, which encodes a sperm-specific protein that compacts sperm DNA. The gene shows a clear excess of function-altering substitutions between humans and chimpanzees (red track) compared to synonymous substitutions, indicative of recurrent positive Darwinian selection occurring over the last millions of years after the split of the two species.
